# TRITEX: chromosome-scale sequence assembly of Triticeae genomes with open-source tools

**DOI:** 10.1186/s13059-019-1899-5

**Published:** 2019-12-18

**Authors:** Cécile Monat, Sudharsan Padmarasu, Thomas Lux, Thomas Wicker, Heidrun Gundlach, Axel Himmelbach, Jennifer Ens, Chengdao Li, Gary J. Muehlbauer, Alan H. Schulman, Robbie Waugh, Ilka Braumann, Curtis Pozniak, Uwe Scholz, Klaus F. X. Mayer, Manuel Spannagl, Nils Stein, Martin Mascher

**Affiliations:** 1 Leibniz Institute of Plant Genetics and Crop Plant Research (IPK) Gatersleben, Seeland, Germany; 2 PGSB – Plant Genome and Systems Biology, Helmholtz Center Munich – German Research Center for Environmental Health, Neuherberg, Germany; 3 Department of Plant and Microbial Biology, University of Zurich, Zurich, Switzerland; 4 Department of Plant Sciences, University of Saskatchewan, Saskatoon, Canada; 5 Western Barley Genetics Alliance, School of Veterinary and Life Sciences (VLS), Murdoch University, Murdoch, WA Australia; 6 Hubei Collaborative Innovation Center for Grain Industry/School of Agriculture, Yangtze University, Jingzhou, China; 7 Department of Agronomy and Plant Genetics & Department of Plant and Microbial Biology, University of Minnesota, St. Paul, MN USA; 8 Green Technology, Natural Resources Institute (Luke), Viikki Plant Science Centre, and Institute of Biotechnology, University of Helsinki, Helsinki, Finland; 9 The James Hutton Institute, Dundee, UK; 10 School of Life Sciences, University of Dundee, Dundee, UK; 11 Carlsberg Research Laboratory, Copenhagen, Denmark; 12 School of Life Sciences Weihenstephan, Technical University of Munich, Munich, Germany; 13 Department of Crop Sciences, Center for Integrated Breeding Research (CiBreed), Georg-August-University Göttingen, Göttingen, Germany; 14 German Centre for Integrative Biodiversity Research (iDiv) Halle-Jena-Leipzig, Leipzig, Germany

## Abstract

Chromosome-scale genome sequence assemblies underpin pan-genomic studies. Recent genome assembly efforts in the large-genome Triticeae crops wheat and barley have relied on the commercial closed-source assembly algorithm DeNovoMagic. We present TRITEX, an open-source computational workflow that combines paired-end, mate-pair, 10X Genomics linked-read with chromosome conformation capture sequencing data to construct sequence scaffolds with megabase-scale contiguity ordered into chromosomal pseudomolecules. We evaluate the performance of TRITEX on publicly available sequence data of tetraploid wild emmer and hexaploid bread wheat, and construct an improved annotated reference genome sequence assembly of the barley cultivar Morex as a community resource.

## Introduction

The Triticeae species wheat and barley were among the founder crops of Neolithic agriculture in Western Asia and continue to dominate agriculture in temperate regions of the world to the present day. Large genome sizes, high content of transposable elements (TEs), and polyploidy (in the case of wheat) have long impeded genome assembly projects in the Triticeae [1, 2]. Recently, chromosome-scale reference sequence assemblies have come available for barley (*Hordeum vulgare*) [3], hexaploid bread wheat (*Triticum aestivum*) [4], and tetraploid durum wheat (*T. turgidum* ssp. *durum*) [5] as well as the wheat wild relatives *Aegilops tauschii* (wheat D genome progenitor) [6], *T. urartu* (wheat A genome progenitor) [7], and *T. turgidum* ssp. *dicoccoides* (wild emmer wheat, AB genome) [8]. The genome projects of barley, bread wheat, and the A and D genome progenitors had initially followed the hierarchical shotgun approach as had been employed by the human genome project [9], but adopted second-generation sequencing methods for sequencing as they became available [10]. Assembling bacterial artificial chromosomes (BACs) guided by a physical map yielded megabase-sized scaffolds [3, 11], which were then arranged into chromosomal super-scaffolds (so-called pseudomolecules) by long-range linkage information afforded by ultra-dense genetic maps [12, 13], chromosome conformation capture sequencing (Hi-C) [14, 15], or Bionano optical mapping [16]. However, BAC-by-BAC assembly is laborious and time-consuming [3] and has become an obsolete method of sequence assembly.

The wild emmer wheat, and subsequently the bread and durum wheat, genome projects [4, 5, 8] used a whole-genome shotgun (WGS) approach based on Illumina short-read sequencing of shotgun libraries with multiple insert sizes. Within months, a fully annotated, highly contiguous sequence was assembled, capturing the full organizational context of the 21 wheat chromosomes, some of which have been validated using other approaches [17]. Despite being robust, the assembly algorithm used in these projects was closed-source [18], potentially limiting its application to the broader community. Indeed, efforts to develop a low-cost, open-source alternative are still required to allow assembly of multiple genomes within a species to comparable contiguity. Short-read assemblies of the wheat genome have been generated by open-source alternatives such as w2rap [19] or Meraculous [13]. In addition, long-read assemblies have been generated for *Ae. tauschii* [20] and bread wheat [21]. But still, the contiguity of these assemblies is lower than that of the scaffolds constructed using the DeNovoMagic algorithm. Another important concern is the high computational cost for a long-read (hybrid) assembly, estimated at 470,000 CPU hours or 6.5 months in wall-clock time [21].

We have recently outlined a proposal for pan-genomics in barley [22]. A cornerstone of our strategy is the construction of high-quality sequence assemblies for multiple genotypes representative of major germplasm groups. Similar projects are under way in bread wheat (http://www.10wheatgenomes.com). An open-source assembly pipeline with comparable accuracy, completeness, and speed similar to available commercial platforms would greatly reduce the cost per assembled genome, thus extending the scope of pan-genome projects in the Triticeae.

Here, we report on the development of a computational pipeline for chromosome-scale sequence assembly of wheat and barley genomes. We evaluate the performance of the pipeline (which we named TRITEX) by re-assembling the raw data used for the wild emmer [8] and bread wheat [4] reference genome assemblies and compare our assemblies to those constructed with a commercial platform. Furthermore, we used TRITEX to generate an improved annotated reference genome assembly for barley cv. Morex as an important resource for the barley research community.

## Results

### Overview of the workflow

We begin with a description of our workflow, its input datasets (Table 1), and a description of the expected outcome of each component (Table 2). For the sake of exposition, we illustrate our method by presenting results for wheat and barley, which will be described in greater detail below.

**Table 1:** Input datasets for TRITEX

Name	Library type (number^1^)	Insert size	Read length	Coverage^2^
PE450	PCR-free paired-end (2)	400–470 bp	2 × 250 bp	70×
PE800	PCR-free paired-end (2)	700–800 bp	2 × 150 bp	30×
MP3	Nextera mate-pair (2)	2–4 kb	2 × 150 bp	30×
MP6	Nextera mate-pair (2)	5–7 kb	2 × 150 bp	30×
MP9	Nextera mate-pair (2)	8–10 kb	2 × 150 bp	30×
10X	10X Chromium (2)		2 × 150 bp	30×
Hi-C	TCC [23] or in-situ Hi-C [24] (1)		2 × 100 bp	200–400 million read pairs

^1^Number of independent libraries to be prepared

^2^Haploid genome coverage for paired-end, mate-pair, and 10X libraries. As Hi-C analysis is count-based, read numbers are more relevant than sequence amount

**Table 2:** Overview of the TRITEX pipeline

	Step^1^	Software	Input	Output
1	Read merging	BBMerge [25]	PE450 read pairs	Merged PE450 reads
2	PE450 error correction	BFC [26]	Merged PE450 reads	Corrected PE450 reads, hash table of k-mer counts
3.1	Unitig assembly	Minia3 [27, 28]	Corrected PE450 reads	Unitigs
3.2	Error correction of PE800 and MP reads	BFC [26], cutadapt [29], NxTrim [30]	PE800, MP3, MP6, and MP9 reads, hash table of k-mer count (step 2)	Corrected PE800, MP3, MP6, and MP9 reads
4	Scaffolding	SOAPDenovo2 [31]	Unitigs; corrected PE800, MP3, MP6, and MP9 reads	Scaffolds
5	Gap-filling	Gapcloser [31]	Scaffolds, corrected PE450 reads	Scaffolds after gap-filling
6.1	Alignment of 10X reads	Minimap2 [32], cutadapt [29], SAMtools [33], BEDtools [34], custom scripts	Scaffolds after gap-filling, 10X reads	10X alignment records
6.2	Alignment of Hi-C reads	As in 6.1, EMBOSS [35]	Scaffolds after gap-filling, Hi-C reads	Hi-C alignment records
6.3	Alignment of genetic markers	Minimap2 [32]	Scaffolds after gap-filling, marker sequences	Marker alignment records
7	Pseudomolecule construction	Custom R scripts	Scaffolds after gap-filling, 10X alignment records, Hi-C alignment records, marker alignment records	Pseudomolecules, Hi-C contact maps

^1^Steps with identical leading digits can be run in parallel

Our pipeline uses the same input datasets generated for DeNovoMagic assemblies reported by Avni et al. [8] and the International Wheat Genome Sequencing Consortium (IWGSC) [4]. The key parameters are two types of paired-end libraries (PE450 and PE800), three types of mate-pair libraries (size ranges 2–4 kb [MP3], 5–7 kb [MP6], and 8–10 kb [MP9]), 10X Chromium libraries, and Hi-C data as listed in Table 1. We show below that certain library types can be omitted in our approach without greatly compromising assembly contiguity.

**Fig. 1:**
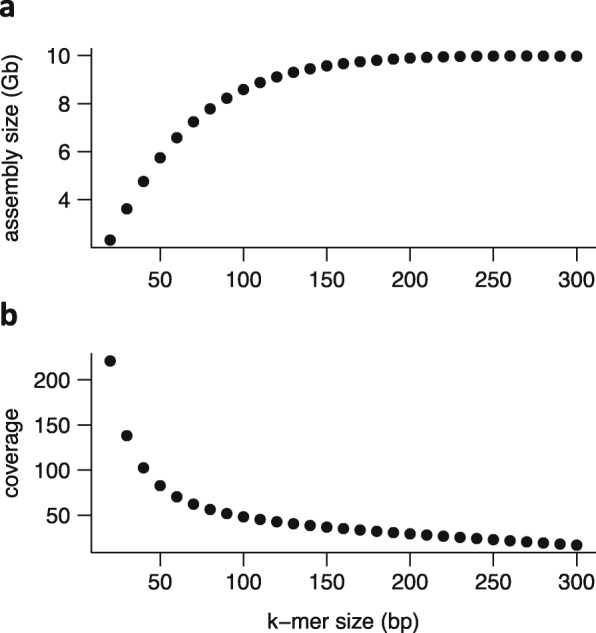
Estimate of assembly size and k-mer coverage as a function of k-mer size. Assembly size (**a**) and k-mer coverage (**b**) were estimated from error-corrected PE450 used for Zavitan unitig assembly based on k-mer cardinalities using NtCard [38] and Kmerstream [37]

A critical component of the “sequencing recipe” are PCR-free Illumina shotgun libraries with a tight insert size distribution in the range of 400–500 bp and sequenced with 250 bp paired-end reads. These were merged with standard tools such as PEAR [36] or BBMerge [25] to yield long single-end reads with a mean fragment size of ~ 450 bp and are subsequently error-corrected with BFC [26]. These elongated short reads allow the use of longer k-mers (i.e., short sequence fragments of fixed length) during the assembly process. Estimates of expected assembly size based on k-mer cardinalities [37] support the notion that longer k-mers achieve much better genome representation in wheat and barley (Fig. 1a). The k-mer size for many assemblers is limited. For example, the maximum k-mer size of SOAPDenovo2 is limited to 127 bp. We thus selected Minia3 [27, 28], an assembler capable of using k-mers of arbitrary size.

A disadvantage of using large k-mer sizes is the lower genome coverage (Fig. 1b) as a consequence of sequencing errors, resulting in random coverage gaps. To overcome this drawback, we adopted the iterative multi-k-mer approach of the GATB-Minia pipeline (https://github.com/GATB/gatb-minia-pipeline). In the initial iteration, an assembly at k-mer size 100 is made from the error-corrected PE450 reads. Subsequent iterations take as input the PE450 reads and assembly constructed in the previous iteration. This procedure is repeated for k-mer sizes 200, 300, 350, 400, 450, and 500. The unitigs of the final iteration achieve an N50 of about 20–30 kb (Table 3). The impact of increasing k-mer size on assembly statistics is summarized in Additional file 1: Table S1. Contig assemblies with smaller k-mer sizes are shorter and hence have a higher proportion of unassembled sequence. This will hamper scaffolding with mate-pair reads as many reads would remain without a mapped mate. An additional advantage of Minia3 over other assemblers such as SOAPDenovo2 [31] or MaSuRCA [39] is its low main memory consumption of only 50 GB. Thus, it is possible to run multiple genomes in parallel, which will be useful in a pan-genome project. A single iteration of Minia3 takes about 1 day for barley and 3 days for hexaploid wheat.

**Table 3:** Assembly statistics for Zavitan and Chinese Spring

	Zavitan		Chinese Spring	
	TRITEX	Avni et al. [8]	TRITEX	IWGSC [4]
Unitig assembly size	10.8 Gb		15.1 Gb	
Unitig N50	21.7 kb		21.4 kb	
Unitig N90	1.5 kb		1.7 kb	
Assembled sequence in contigs ≥ 1 kb	10.0 Gb		14.0 Gb	
Assembled sequence in contigs ≥ 10 kb	7.8 Gb		10.8 Gb	
Scaffold assembly size	11.1 Gb	10.5 Gb	15.7 Gb	14.5 Gb
Scaffold N50	1.3 Mb	7.0 Mb	2.3 Mb	7.0 Mb
Scaffold N90	97 kb	1.2 Mb	281 kb	1.2 Mb
Assembled sequence in scaffolds ≥ 1 kb	10.4 Gb	10.5 Gb	14.8 Gb	14.5 Gb
Assembled sequence in scaffolds ≥ 1 Mb	6.7 Gb	9.6 Gb	11.9 Gb	13.4 Gb
Unfilled internal gaps	209 Mb (1.9%)	171 Mb (1.6%)	476 Mb (3.0%)	262 Mb (1.8%)

The unitigs of the final iteration are used as input for scaffolding with the PE800, MP3, MP6, and MP9 libraries using SOAPDenovo2 [6]. We have also evaluated two other tools (BESST [40], OperaLG [41]), but only BESST ran successfully on our dataset. BESST yielded assembly of lower quality than SOAPDenovo2 (Additional file 1: Table S2) and had longer runtimes. Scaffolding with SOAPDenovo2 yields assemblies with an N50 beyond 1 Mb (Tables 3 and 4). After gap-filling with GapCloser, about 1–5% internal gaps in scaffolds remain (Tables 3 and 4). Alignments of 10X and Hi-C reads and genetic markers to the scaffolds are imported into R [43], and custom scripts were developed to identify and correct mis-assemblies, to construct super-scaffolds, and to build pseudomolecules. Both super-scaffolding with 10X data and pseudomolecule construction use the POPSEQ genetic maps of barley [12] and wheat [13] to guide the assignment of scaffolds to chromosomes and to discard spurious links between unlinked regions. We note that the omission of the PE800, MP3, and MP6 libraries (i.e., using only the MP9 library for mate-pair scaffolding) resulted in assemblies of comparable contiguity and genome representation in barley (Table 4). If this slightly reduced contiguity is acceptable for downstream application, the cost for data generation can be reduced by about 20%.

**Table 4:** Comparison of different assemblies of barley cv. Morex

	BAC-by-BAC		TRITEX	TRITEX
	Morex V1 [3]	Dovetail	Morex V2	MP9 only
Scaffold assembly size	4.79 Gb		4.65 Gb	4.6 Gb
Scaffold N50	79 kb		3.4 Mb	2.6 Mb
Scaffold N90	4.4 kb		287 kb	150 kb
Assembled sequence in scaffolds ≥ 1 kb	4.67 Gb		4.34 Gb	4.32 Gb
Assembled sequence in scaffolds ≥ 1 Mb	0 bp		3.80 Gb	3.49 Gb
Unfilled internal gaps	216 Mb (4.5%)		116 Mb (2.5%)	106 Mb (2.3%)
Super-scaffold N50	1.9 Mb	1.3 Mb	40.2 Mb	32.6 Mb
Super-scaffold N90	336 kb	7.5 kb	2.0 Mb	1.2 Mb
Size of pseudomolecules	4.58 Gb		4.26 Gb	4.20 Gb
Size of unanchored sequences (chrUn)	246 Mb		83 Mb^2^	111 Mb^2^
Proportion of complete full-length cDNAs^1^	81.8%	84.1%	89.8%	90.4%

^1^Proportion of 28,622 full-length cDNAs of barley cv. Haruna Nijo [42] aligned with ≥ 90% coverage and ≥ 97% alignment identity

^2^Sequences shorter than 1000 kb were not included in chrUn

**Fig. 2:**
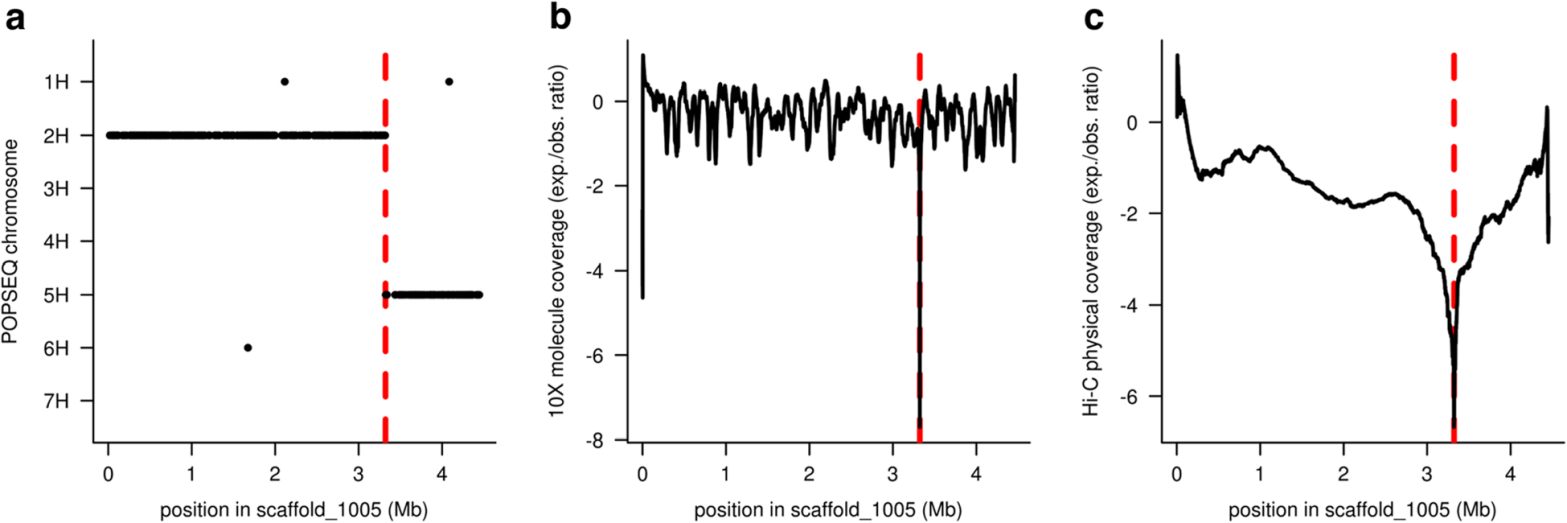
Example of a chimeric scaffold. The chimeric nature of a sequence scaffold joining two unlinked sequences originating from barley chromosomes 2H and 5H is supported by multiple lines of evidence. **a** Genetic chromosome assignments of marker sequences aligned to scaffold_1005. **b** 10X molecule coverage. **c** Physical Hi-C coverage. Coverage in **b** and **c** was normalized for distance from the scaffold ends and the log2-fold observed vs. expected ratio was plotted. The red, dotted lines mark the breakpoint at 3.32 Mb

**Fig. 3:**
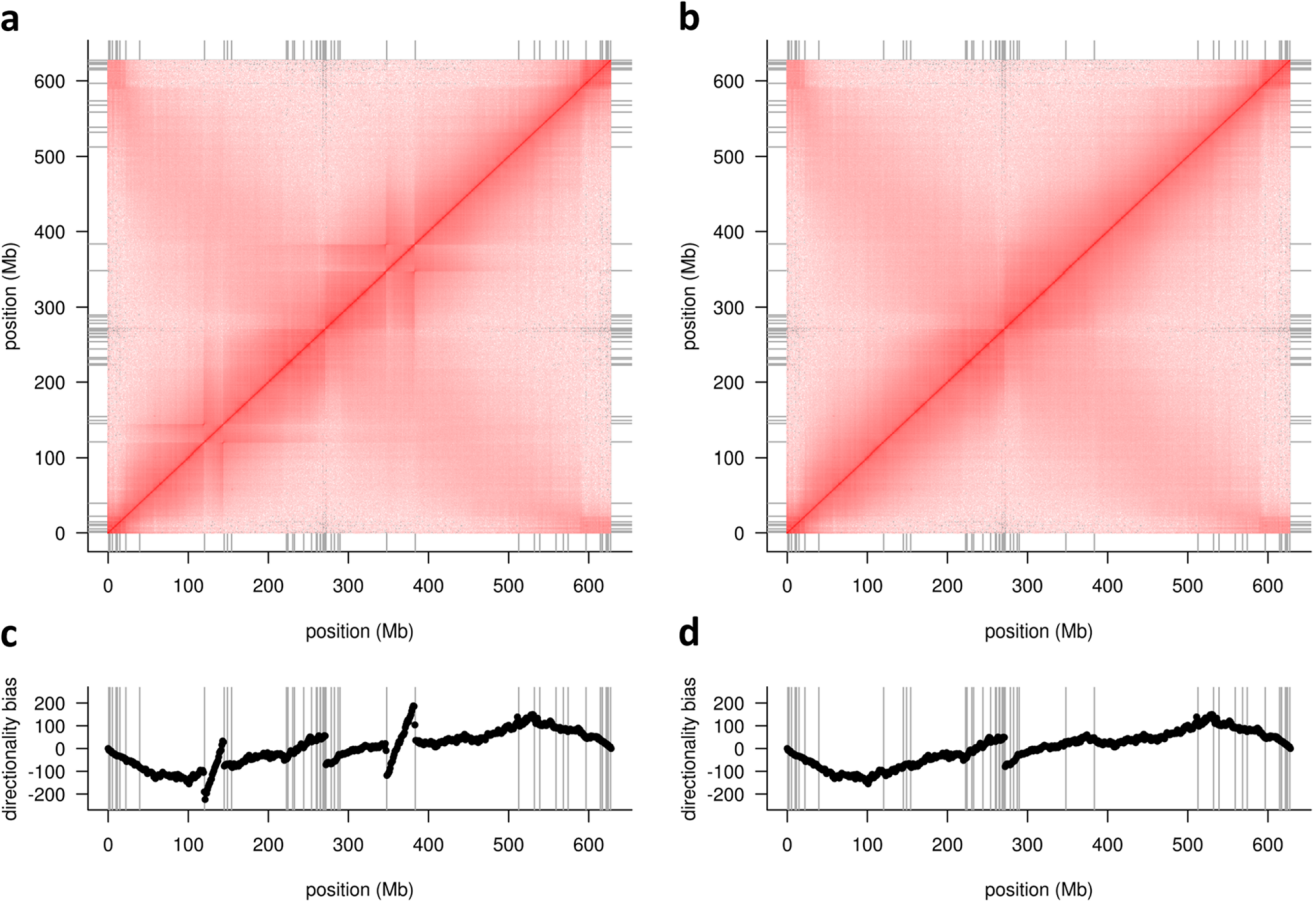
Example of errors in scaffold orientation. The top panels show the Hi-C contact matrix for barley chromosome 3H before (**a**) and after (**b**) manual correction. The bottom panels show the directionality biases in the Hi-C data as defined by Himmelbach et al. [47] before (**c**) and after (**d**) manual correction. Two inverted scaffolds are evident as deviations from the expected Rabl configuration [3] and as diagonals bounded by discontinuities in the directionality biases

Scaffolding can introduce false joins between unlinked sequences [44] that need to be broken to construct correct chromosomal pseudomolecules [45]. Physical coverage with 10X reads is used to detect and correct mis-joins introduced during either unitig construction or scaffolding (Fig. 2). The corrected scaffolds are used as input for super-scaffolding with 10X data using a custom graph-based method (see the “Methods” section for details). These super-scaffolds are then ordered and oriented along the chromosomes using Hi-C data using the method of Beier et al. [46]. Once scaffolds have been arranged into chromosomal pseudomolecules, contact matrices for each chromosome are plotted as heat maps. Visual inspection of these matrices can reveal further assembly errors such as remaining chimeras or misoriented (blocks of) super-scaffolds (Fig. 3). After correction of these errors, the Hi-C maps are updated and cycles of assembly-inspection-correction are repeated until all mis-assemblies have been eliminated and contact matrices show the expected Rabl configuration [3] (strong main diagonal/weak anti-diagonal). We found that pseudomolecules constructed from corrected super-scaffolds contain in the range of 10 to 20 mis-assemblies, which were eliminated in a single correction cycle. Without 10X data, i.e., using only Hi-C data for spotting mis-assemblies as in the case of published wheat reference genomes [4, 8], more curation cycles were required.

Assuming all input datasets (Table 1) are in place, the entire TRITEX workflow can be completed in 3 to 4 weeks for barley and 4 to 6 weeks for hexaploid wheat, allowing for some delays in the completion of hands-on steps (mainly inspection of intermediate results and curation of pseudomolecules). We believe that despite our detailed user guide (available at https://tritexassembly.bitbucket.io), completing a TRITEX assembly would be a rather arduous task for a scientist inexperienced in either plant genome assembly or practical bioinformatics, unless guided by an expert in plant genomics. A UNIX server with at least 1 TB of main memory is needed to complete scaffold construction for bread wheat. Much wall-clock time (about 1 week for barley [5 Gb genome]) and about 3 weeks for bread wheat [16 Gb genome]) is spent for unitig assembly with Minia3. Fortunately, the main memory consumption of Minia3 is low (50 GB). Thus, assemblies of multiple genotypes (a typical use case in a pan-genome project) can be run in parallel.

### Re-assembly of wild emmer and bread wheat and comparison to published assemblies

**Fig. 4:**
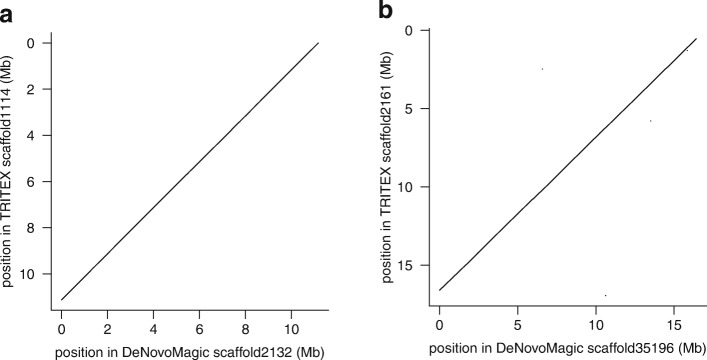
Collinearity between TRITEX and DeNovoMagic assemblies of wheat**.** Dot plots showing the longest alignments between scaffold pairs of the TRITEX and DeNovoMagic assemblies of Zavitan (**a**) and Chinese Spring (**b**), respectively. Alignments were done with Minimap2 [32]

We downloaded the paired-end and mate-pair reads used for the DeNovoMagic assemblies of wild emmer wheat accession Zavitan [8] and bread wheat cultivar Chinese Spring [4] (referred to as the IWGSC whole-genome assembly in Table S2 of [4]) and ran TRITEX until the gap-filling step (step 2 in Table 2). The metrics of TRITEX assemblies were inferior to those of DeNovoMagic (Table 3). Still, the contiguity of the TRITEX assemblies was in the megabase range, and it was clearly superior to the BAC-by-BAC assembly of a single wheat chromosome (3B [11], N50: 892 kb). Visual inspection of alignments between the TRITEX and DeNovoMagic assemblies indicated a high concordance between them (Fig. 4). To assess the accuracy of the TRITEX assemblies at a genome-wide scale, we compared the TRITEX scaffolds to published assemblies produced by the DeNovoMagic algorithm. These assemblies had been independently validated by complementary sequence and mapping resources [4, 8, 48]. We divided the TRITEX scaffolds into non-overlapping 10 kb fragments, aligned them to the DeNovoMagic assemblies with Minimap2 [32], and measured the collinearity of the alignments. The average Pearson correlation of fragment positions in the TRITEX scaffold and their aligned positions in the DeNovoMagic scaffolds was 0.998 for Chinese Spring and 0.999 for Zavitan. Across all Chinese Spring (Zavitan) scaffold pairs with at least 100 kb of aligned sequences, 99.96% (99.99%) of aligned fragment sequences were mapped in the same orientation. These results support a very high concordance in the local order and orientation of sequences between the TRITEX and previous assembly efforts.

To assess the completeness of the TRITEX assembly of Chinese Spring, we determined the representation of two transcript resources: the IWGSC gene models [4] and the full-length cDNAs of Mochida et al. [49]. The proportion of completely represented transcripts in the TRITEX assembly was very similar to the IWGSC RefSeq and substantially higher than in the w2rap assembly [19] and the PacBio hybrid assembly of Zimin et al. [21] (Table 5). Note that the IWGSC RefSeq gene models are likely to have a bias for the TRITEX assembly, which was generated from the same input data, but it is not evident how the Sanger-sequenced full-length cDNAs might favor a certain assembly.

**Table 5:** Chinese Spring transcript alignment statistics

Transcript dataset	No. of transcripts	Assembly	Proportion of complete transcripts^1^ (%)
IWGSC v1.0 transcripts [4]	269,583	TRITEX	96.2
		IWGSC [4]	97.0
		Clavijo et al. [19]	87.8
		Zimin et al. [21]	88.5
Full-length cDNAs [49]	6137	TRITEX	97.1
		IWGSC [4]	96.3
		Clavijo et al. [19]	91.6
		Zimin et al. [21]	85.4

^1^Proportion of transcripts with at least one alignment with ≥ 99% coverage and ≥ 99% identity (for IWGSC transcripts) or with ≥ 90% coverage and ≥ 99% identity (for full-length cDNAs). Alignments were done with GMAP [50]

A comparison of the TRITEX assemblies of Zavitan and Chinese Spring to their published counterparts revealed a higher proportion of sequence gaps (Table 3). We speculate that sequence gaps may arise because highly similar copies of transposable elements (TEs) cannot be resolved. To test this hypothesis, we analyzed the representation of two TE families, RLC_Angela and RLC_Sabrina [51], in the TRITEX and IWGSC WGA assemblies of Chinese Spring. Despite having similar assembly sizes (Table 3), we identified substantially fewer full-length RLC_Angela elements in the TRITEX assembly, whereas the numbers of RLC_Sabrina elements matched closely in both assemblies (Additional file 1: Table S3). RLC_Angela is considered a recently active (i.e., transposing) family, whereas RLC_Sabrina has been inactive for a long time (Thomas Wicker, unpublished results). Consistent with the expectation that younger elements, which inserted recently and have highly similar copies elsewhere in the genomes, are not well assembled, the age distribution of RLC_Angela is skewed for older elements. By contrast, no such bias is seen for RLC_Sabrina (Additional file 1: Figure S1). In summary, the TRITEX assembly of Chinese Spring has fewer complete TEs, indicating that the DenovoMagic algorithm may make better use of mate-pair or PE450 data to close gaps in TEs.

### An improved barley reference genome assembly

Prompted by the encouraging assembly results for wheat, we decided to employ the TRITEX pipeline to construct a second version reference genome assembly of barley cv. Morex. The need for an improved assembly arose from shortcomings of the BAC-based reference sequence [3] including (1) large sequence gaps, (2) redundancies, and (3) local mis-assemblies.

First, gaps in the physical map or failed BAC assemblies result in gaps in the assembled sequence that may contain important genes. During the process of pseudomolecule construction [46], we attempted to “rescue” missed genes by adding sequences from a WGS draft assembly of Morex [52]. However, the short WGS contigs do not provide the local sequence context of genes and may not even contain full-length gene sequences. Second, it was necessary to merge sequences from individually assembled BAC clones [10] during pseudomolecule construction [46]. Megabase-sized sequence scaffolds representing physical contigs of BACs were constructed using a complex, multi-tiered method that employed heuristic approaches to distinguish true sequence overlaps from alignments caused by highly similar copies of transposable elements [46]. Nevertheless, self-alignment of the pseudomolecule at a high identity threshold (minimum alignment length, 5 kb; minimum alignment identity, 99.5%) resulted in a substantial proportion (4.4%) of undetected overlaps between adjacent BACs. Similar results were obtained for the first version of the BAC-based maize reference genome [53] (1.2%) and the 3B pseudomolecule of Choulet et al. [11] (7.4%) using the same alignment thresholds.

Third, individual BAC clones were rarely represented by a single sequence scaffold even after scaffolding with mate-pair data [10]. At the time barley BAC sequencing was performed, methods with a sufficient density and resolution to order and orient sequence scaffolds within 100 kb were not available. Hence, our solution [46] was to place sequence scaffolds originating from the BAC clone in arbitrary order and orientation into the pseudomolecule, thus introducing many local assembly errors at the sub-BAC scale.

Our results in wheat led us to expect that a TRITEX assembly of the Morex genome would overcome the limitations inherent to the BAC-by-BAC approach. To construct a TRITEX assembly of the Morex genome, we obtained the datasets as detailed in Table 1. New paired-end, mate-pair, and 10X libraries were constructed and sequenced. The Hi-C data of Mascher et al. [3] were used for pseudomolecule construction. The assembly metrics of the Morex TRITEX assembly greatly exceeded those of the BAC-by-BAC assembly. Notably, the proportion of completely aligned full-length cDNAs improved by about nine percentage points (Table 4) compared to the BAC-by-BAC assembly.

To ascertain the correct local order and orientation of sequence scaffolds, we compared the TRITEX super-scaffolds to three complementary resources: (i) the first version (V1) pseudomolecules, (ii) the BAC-by-BAC assembly improved by super-scaffolding based on newly collected in vitro proximity ligation, and (iii) the genome-wide optical map of Morex.

**Fig. 5:**
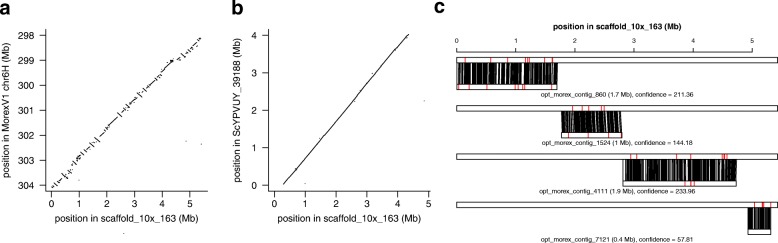
Morex V2 assembly validated by complementary resources. Morex scaffold_10x_163 was aligned to the Morex V1 assembly (**a**), the Dovetail assembly of Morex (**b**), and the genome-wide optical map of Morex (**c**). Sequence alignments are shown as dot plots (**a**, **b**). **c** The alignment of four optical contigs to scaffold_10x_163. Single aligned restriction sites are connected by black lines. Red lines indicate unaligned restriction sites in either the sequence scaffold or the optical contig

First, visual inspection of alignments confirmed the expected discordances at the sub-BAC level, but showed good collinearity at the megabase-scale (Fig. 5a). We used the same approach as for the comparison to the published wheat assemblies by aligning 10 kb fragments. The Pearson correlation between TRITEX fragments and their aligned positions in the Morex V1 pseudomolecules was 0.927, reflecting a breakdown of collinearity at finer resolution. The orientations in the pseudomolecules of fragments originating from the TRITEX super-scaffolds were highly discordant: on average, only 63% of aligned sequence in TRITEX/Morex V1 scaffold pairs was in concordant orientation.

Second, we compared the TRITEX super-scaffolds to an improved version of the BAC-by-BAC assemblies of Mascher et al. [3]. Before the development of TRITEX, we had attempted to order and orient BAC sequence scaffolds using the Dovetail method. This involved in vitro proximity ligation sequencing (Chicago) followed by scaffolding with the HiRise assembler [54]. Visual inspection of alignments between TRITEX super-scaffolds and Dovetail scaffolds revealed a higher concordance compared to the V1 pseudomolecules (Fig. 5a, b). At the level of 10-kb fragment alignments, the collinearity between TRITEX positions and mapped position in the Dovetail assembly was 0.982 (Pearson correlation). On average, 94.8% of aligned sequence in TRITEX/Dovetail scaffold pairs was concordant orientation. We note that the Dovetail assembly was based on the same sequence scaffolds generated from single assembled BAC clones as were used in the Morex V1 pseudomolecule. Hence, the issues of sequence gaps due to failed BAC assemblies and artificial duplication persist. Nevertheless, Dovetail scaffolding did improve the presentation of complete full-length cDNAs by about two percentage points (Table 4), most likely by mending occasional sequence breaks within genes.

Third, we compared the TRITEX super-scaffolds to the optical map of the Morex genome constructed by Bionano genome mapping [3, 16]. The optical contigs were aligned to the in silico digested TRITEX assembly using Bionano’s Refaligner. Of Nt.BspQ1 sites in the assembly, 95.9% were covered by high-confidence alignments (score ≥ 20) of optical contigs and 88.6% of label sites were aligned. Vice versa, 95.3% of label sites in the Bionano map were spanned by high-confidence alignments and 90.0% of Bionano label sites were aligned to the sequence assembly. Label sites covered by alignments, but themselves not aligned (red lines in Fig. 5c) may be due to missed label sites in the optical map, gaps in the sequence assembly, or alignment uncertainties. We note that it was not possible to align optical contigs to the BAC-based sequence scaffolds as their contiguity is too low (N50: 79 kb, Table 4, [46]). In summary, all three comparisons support the high local accuracy of the TRITEX assembly.

**Fig. 6:**
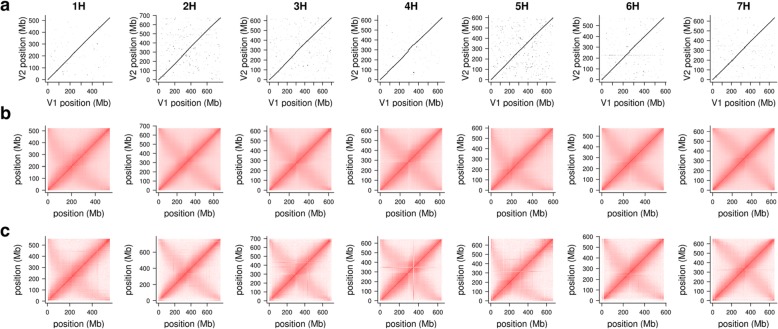
Collinearity of Morex V1 and V2 assemblies. **a** Dot plots showing the alignments between the chromosomal pseudomolecules of the Morex V1 and V2 assemblies. **b** Intra-chromosomal Hi-C contact matrices of the Morex V2 assembly. **c** Intra-chromosomal Hi-C contact matrices of the Morex V1 assembly

To assess the accuracy at the pseudomolecule level, we plotted alignments between chromosomal pseudomolecules of Mascher et al. ([3], Morex V1) and those constructed using TRITEX (Morex V2) and inspected Hi-C contact matrices (Fig. 6). The V1 and V2 pseudomolecules were highly collinear. The contact matrices showed the expected Rabl pattern. Several smaller mis-assemblies present in the V1 pseudomolecule were corrected in V2. For example, Morex V1 had a misplaced sequence in the peri-centromeric regions of chromosome 4H (300–400 Mb, Fig. 6c), which was correctly placed in Morex V2 as supported by the Hi-C contact matrix (Fig. 6b).

The amounts and characteristics of repetitive sequences such as TEs and tandem repeats represented in sequence assemblies can serve as proxies for assembly quality. We compared the Morex V1 and V2 assemblies according to five criteria: (i) overall TE composition, (ii) presence of highly abundant 20-mers, (iii) amount and localization of tandem repeats, (iv) the amount and age distribution of retrotransposons, and (v) sequence gaps in selected TE families. Overall TE composition was almost identical between the assembly versions (Additional file 1: Table S4). The chromosomal distribution of highly abundant 20-mers was similar in the Morex V1 and V2 assemblies, but Morex V2 contains more repetitive sequence in peri-centromeric regions (Additional file 1: Figure S2a). Similarly, the Morex V2 assembly contains 50% more tandem repeats than V1. Notably, the number of satellite tandem repeats is almost doubled. Tandem repeats are concentrated in short sequence scaffolds not assigned to chromosomes (chrUn) in Morex V1 and at distal ends of several chromosomes (short arm of 4H, long arms of 4H and 6H) and in peri-centromeric regions (Additional file 1: Figure S2b).

The representation of long terminal repeat (LTR) retrotransposon families was similar in both assemblies (Additional file 1: Table S4). However, the Morex V2 assembly contains 1590 (5%) more intact full-length elements than V1 (Additional file 1: Table S5, Figure S3a, b). In both assemblies, the number of retrieved full-length LTRs matches the expectation based on genome size (Additional file 1: Figure S3c). Insertion age distributions show that the Morex V2 assembly resolved a higher number of younger *Copia* elements (Additional file 1: Figure S3d). The distinct peak at age 0 in the V1 assembly is most likely caused by a scaffolding artifact from the chromosomal pseudomolecule construction when sequences from the same BAC were arranged in arbitrary order in the Morex V1 pseudomolecules as described above. To understand the impact of sequence gaps on TE representation in the Morex V2 assembly, we performed a similar analysis as for Chinese Spring, using the recently active BARE1 family. The Morex V2 assembly contains more full-length elements than V1 (5471 vs. 3469; Additional file 1: Table S3). Moreover, the percentage of full-length copies that are flanked by a target site duplication (TSD) is higher in the V2 assembly (90% vs. 81%), suggesting fewer chimeric sequences. However, the size distribution of the elements in the Morex V2 assembly indicates a large population of overly large full-length elements (Additional file 1: Table S6). In contrast, the size distribution of full-length elements in Morex V1 is narrower and shows two characteristic peaks corresponding to the autonomous and non-autonomous subfamilies (T. Wicker, unpublished results). Manual inspection of 50 randomly selected elements between 9900 and 10,000 bp in length showed that the large sizes of these elements are mainly due to large sequence gaps (i.e., long stretches of N’s). In the 50 manually inspected copies, we found 70 sequence gaps in the internal domain and only 5 short gaps in the LTRs. The latter observation is not surprising as our method to identify full-length copies relied on largely gap-free LTRs. In only three cases, the large size of the element was caused by the genuine insertion of additional TEs. Overall, the Morex V2 assembly had more and larger gaps as TE length increased (Additional file 1: Table S6), a pattern that is absent from the Morex V1 assembly. In summary, the representation of repetitive sequence is similar in both assembly versions of the Morex genome. The longer read lengths and k-mer sizes used in the TRITEX pipeline may have resulted in a better representation of short tandem repeats in V2. However, the gap-free assembly of very recently inserted full-length TEs may benefit from prior complexity reduction such as BAC sequencing.

To facilitate the adoption of the Morex V2 assembly as a common reference sequence by the cereal research community, we annotated the pseudomolecules using the same transcript datasets as used by Mascher et al. [3] for Morex V1, but with an improved version of the PGSB annotation pipeline. A total of 32,787 high-confidence (HC) and 30,871 low-confidence (LC) gene models were annotated on the V2 pseudomolecules. Of the 1440 BUSCOs (Benchmarking Universal Single-Copy Orthologs, [55]), 98.9% were completely represented by annotated genes, a 6.4% increase compared to the V1 annotation. At the same time, the V2 annotation has fewer high-confidence gene models (32,787 [V2] vs. 39,734 [V1]), likely owing to higher assembly contiguity (i.e., fewer fragmented gene models), more stringent thresholds during the annotation process, and the incorporation of TE annotations as hints for ab initio prediction to reduce the number of transposon-related genes. In a comparison against an independent reference database comprising a curated protein set from 11 grass species, the Morex V1 protein sequences were on average shorter than their V2 counterparts as indicated by a lower alignment coverage. An analysis of sequence gaps in the intergenic space surrounding genes revealed that 90% of V2, but only 60% of V1, genes models do not have any “N” bases in their 1-kb flanking regions in the respective sequence assemblies (Additional file 1: Figure S4). Thus, the Morex V2 gene annotation represents more complete gene models and more regulatory regions around genes compared to the V1 annotation. In conclusion, the TRITEX assembly of Morex constitutes a greatly improved barley reference genome and will serve as an important community resource.

## Discussion

We have developed an open-source pipeline for chromosome-scale sequence assemblies of wheat and barley, and validated its performance by comparison to complementary sequence and mapping resources available for the two species. We believe the main application of TRITEX will be in (i) cereal pan-genomics (i.e., assembling genome sequences for representative genotypes), (ii) phylogenomics (i.e., assembling crop-wild relatives in the Triticeae), and (iii) gene isolation (assisting map-based cloning projects) in the immediate future.

First, in a pan-genomics scenario, it is desirable to achieve chromosome-scale sequences of a (few) dozen genotypes representative of major germplasm groups [22]. An open-source pipeline provides the cereal genomics community with a cost-effective platform to generate comparable genome sequences that are amendable to further improvement and refinement. The up-front cost of purchasing hardware (or leasing cloud computing) and (self-) educating researchers in assembly methodology is justified if many assemblies are done. As service fees for assembly may be as high as the expenses for data generation, academic researchers can double the number accessions included in a pan-genomics project if they perform sequence assembly on their own. Alternative on-site computing infrastructures are national computing infrastructures such as CyVerse [56], de.NBI [57], or SNIC [58].

Second, we anticipate that TRITEX will work well in any diploid or allopolyploid inbreeding Triticeae species. We and others used TRITEX to construct chromosome-scale pseudomolecules for the diploid wheat wild relatives *Aegilops sharonensis* and *Ae. longissima*, two important donors of disease resistances [59, 60]. The super-scaffold N50 values were 12.3 Mb for *Ae. sharonensis* (Guotai Yu and colleagues; unpublished results) and 3.8 Mb for *Ae. longissima* (Raz Avni, Assaf Distelfeld, Amir Sharon; unpublished results). A total of 6.6 Gb of sequence were assembled into chromosomal pseudomolecules in both species (expected genome size, ~ 7.5 Gb). Our pipeline should be applicable to rye (*Secale cereale*), a minor cereal crop with great importance in East and Central Europe. Although rye is a highly heterozygous, outcrossing species, inbred lines are frequently used by breeders and genomic researchers [61, 62]. TRITEX is likely to work well in species with large and/or allopolyploid genomes outside the Triticeae tribe such as maize and oats. We evaluated the performance of TRITEX on one hexaploid oat genotype (*Avena sativa*) and achieved scaffold N50 values of 1.5 Mb (Tim Langdon; unpublished results). Comparison to genetic maps confirmed that TRITEX is able to separate homeologs in allohexaploid oat as it can do in wheat. We have not run TRITEX for maize, but high-quality sequence assemblies have been constructed with a commercial assembly algorithm [63–65] on short-read data compatible with the TRITEX requirements. However, we must caution users that TRITEX yields assemblies of much lower contiguity and genome representation if closely related haplotypes resident in the same nucleus have to be resolved (Bruno Studer, unpublished results). Therefore, we encourage researchers aiming at assembling the genomes of heterozygous, autoploid, or dikaryotic species to use long-read sequencing.

Third, Thind et al. [66] recently used chromosomal genomics to assist a gene isolation project. They constructed a megabase-scale sequence scaffold of wheat chromosome 2D of cultivar CH Campala harboring flanking markers of a leaf rust resistance locus to isolate a candidate gene, which was absent from the Chinese Spring reference. In cases, where flow-sorting is not possible, the same purpose (albeit at a higher cost) may be served by TRITEX whole-genome assemblies of the parents of a mapping population or near-isogenic lines carrying mutant introgressions. De novo sequence assembly may be of particular relevance to understanding the molecular basis of plant performance in crop-wild introgression lines derived from wide crosses harboring introgressed segments highly divergent from the reference sequence of the domesticate. For example, these might be pre-breeding material with improved disease resistance, but suffering from linkage drag [67], or released wheat cultivars with alien introgressions conferring superior agronomic performance [68].

The modular layout of our pipeline lends itself to improvement, replacement, or simplification of its components. An integral component of the pipeline is the application of Minia3 to make best use of the PE450 data. Other tools may use standard task, for example, Trimmomatic [69] instead of cutadapt [29] for read trimming or Bloocoo [70] instead of BFC [26] for error correction. Compared to DeNovoMagic, TRITEX achieves a lower contiguity at the scaffold level and has a higher proportion of internal gaps (Tables 3 and 4). Frequent gaps and breaks in repetitive sequence are inherent to short-read assemblies. In fact, long-read assemblies with *contig* N50s exceeding *scaffold* N50s of short-read assemblies have been obtained in other plant and animal species [71–73]. Thus, we propose to improve our Illumina-based scaffolding and gap-filling methodology by integrating long-read sequencing into TRITEX in the future. This can be accomplished either by replacing contigging and mate-pair scaffolding entirely with long-read assembly—contingent on the feasibility of obtaining megabase-scale contig N50s in the Triticeae. Alternatively, contig assembly with PE450 reads may be maintained, but long-read sequences could be used for scaffolding and closing gaps. Depending on their accuracy and relative cost- and time-effectiveness, both approaches may be valid for different applications. Long-read sequencing in the Triticeae may adopt the recently developed circular consensus method of Wenger et al. [74] or improved Nanopore sequencing to obtain highly accurate long reads. These will likely be crucial to resolve homeologs in polyploid wheat where genic sequence divergence between subgenomes is lower than the error rate of uncorrected long reads.

Our methods for pseudomolecule construction evolved from scripts used for Hi-C mapping of BAC-based sequence scaffolds of barley [3] and whole-genome shotgun assemblies of wheat [4, 8]. They can correct, order, and orient along the chromosome sequence scaffolds of sufficient contiguity and genome representation produced by any sequencing and assembly strategy. We anticipate that even the best long-read assemblies will not be error-free and yield chromosomal contigs without the use complementary linkage information Thus, our methods for assembly correction and super-scaffolding based on linked-read and Hi-C will likely survive the transition to long reads for contig assembly.

For some research purposes, chromosomal sequences may not be required. If a narrow target interval has been defined in a map-based cloning project, PE450 and MP9 reads may suffice to obtain a single sequence scaffold harboring flanking markers at both sides. In this case, Hi-C and even 10X sequencing can be forgone for faster and cheaper assembly, but at the expense of sequence contiguity.

## Methods

### High molecular weight DNA extraction

High molecular weight (HMW) DNA depleted for plastidal genomes were prepared from fresh leaves of 1-week-old seedlings of “Morex” using a large-scale phenol:chloroform extraction [8]. In short, the protocol involves isolation of nuclei from fresh leaf material, then the nuclei are treated with proteinase-K, and phenol-chloroform extraction removes protein contamination. Then, the HMW DNA was spooled out of the solution using sodium acetate and ethanol precipitation. The extracted DNA was used for preparation of PCR-free paired-end libraries, mate-pair libraries with specific insert sizes and 10X Chromium libraries.

### Library preparation and sequencing

PCR-free paired-end libraries with 400–500 bp insert sizes (PE450) for barley cv. Morex were prepared using a custom protocol using the Illumina Truseq PCR-free library preparation kit. The protocol starts with fragmentation of HMW DNA by ultrasound (Covaris S220, duty factor 8%, peak incident power 160, cycles per burst 200, time 60 s) followed by BluePippin size selection on a 1.5% cassette with tight 470 bp setting. Then, the size-selected DNA was used as input material for Truseq DNA PCR-free library preparation without the SPB bead-based size selection. The prepared libraries were quantified using the KAPA library quantification kit. Sequencing of the PE450 libraries was done on the HiSeq2500 system in Rapid Run mode (2 × 266 bp reads). The Morex PE800 paired-end library (insert size range, 700–800 bp) as well as MP3 (insert size range, 2–4 kb), MP6 (5–7 kb), and MP9 (8–10 kb) mate-pair libraries was constructed and sequenced (2 × 150 bp reads) at the University of Illinois Roy J. Carver Biotechnology Center.

To prepare 10X genomics libraries, genomic DNA (gDNA) was quantified by fluorometry (Qubit 2.0). Small fragments (< 40 kb) were removed from ~ 2 μg of gDNA using pulsed-field electrophoresis on a Blue Pippin instrument (Sage Science, http://www.sagescience.com) following the high-pass protocol. Recovered HMW DNA was evaluated for integrity and size (> 48.5 kb) on a Tapestation 2200 (Agilent, https://www.agilent.com), and quantified (Qubit 2.0, https://www.thermofisher.com/de/en/home/industrial/spectroscopy-elemental-isotope-analysis/molecular-spectroscopy/fluorometers/qubit.html). Library preparation followed the 10X Genome Chromium library protocol v1 (10X Genomics, https://www.10xgenomics.com). Four individual libraries were prepared and uniquely indexed for multiplexing, and quantified by qPCR (Kapa Biosystems). Libraries were normalized and pooled for sequencing on two lanes of an Illumina HiSeq2500 instrument in PE125 mode using v4 chemistry for high output. Pooled libraries were de-multiplexed with Supernova (10X Genomics), and FASTQ files generated with Longranger (10X Genomics).

### Preprocessing of paired-end and mate-pair reads

Overlapping single reads of the PE450 libraries were merged with PEAR [36] (Zavitan) or with BBMerge [25] (Chinese Spring, Morex) using the “maxloose” strictness setting. Error correction of merged PE450 reads was done with BFC [26] in two passes. After the first BFC pass (correction), reads containing singleton k-mers were trimmed using a k-mer size of 61. Illumina adapters were trimmed from the PE800 read using cutadapt [29]. Nextera junction adapters and short-insert contaminants were removed from mate-pair reads using NxTrim [30]. Trimmed PE800 and mate-pair reads were corrected with BFC [26] using the hash table of k-mer counts generated from the PE450 reads.

### Unitig assembly

Minia3 ([28], https://github.com/GATB/minia) was used to assemble corrected and trimmed PE450 reads into unitigs. The Minia3 source was assembled to enable k-mer size up to 512 as described in the Minia3 manual. The parameters “-no-bulge-removal -no-tip-removal -no-ec-removal” were used to disable the resolution of ambiguous paths. Iterative Minia3 runs with increasing k-mer sizes (100, 200, 300, 350, 400, 450, 500) were used as proposed in the GATB Minia pipeline (https://github.com/GATB/gatb-minia-pipeline). In the first iteration, the input reads were assembled using a k-mer size of 100. In the subsequent runs, the input reads as well as the assembly of the previous iteration were used as input for the assembler.

### Scaffolding and gap closing

Error-corrected PE800, MP3, MP6, and MP9 reads were used for scaffolding with SOAPDenovo2 [31]. The “fusion” module (https://github.com/aquaskyline/SOAPdenovo2) was used to prepare the Minia3 unitigs for use with SOAPDenovo2. The “map” was used to align reads to the unitigs. A range of parameters for “pair_num_cutoff” (minimum of read pairs linking two sequences) for each library were tested with the “scaff” module with gap-filling disabled, and the “pair_num_cutoff” value resulting in the best N50 was chosen. Once the best thresholds had been determined, the “scaff” module was run with gap-filling enabled (parameter -F).

GapCloser (https://sourceforge.net/projects/soapdenovo2/files/GapCloser/src/r6/) was used to fill internal gaps in scaffolds using the error-corrected PE450 reads.

### Alignment of 10X Chromium reads and molecule calling

Before alignment, the FASTQ files of read 1 and read 2 were interleaved with Seqtk (https://github.com/lh3/seqtk). Then, the first 23 nt of read 1 were removed and the instrument name in the Illumina read identifier was replaced by the 10X barcode (the first 16 nt of read 1). Illumina adapters were trimmed using cutadapt [29] using the adapter sequence AGATCGGAAGAGC. Reads shorter than 30 bp after trimming were discarded. Trimmed reads were aligned to the assembly after the GapCloser step using Minimap2 [32]. Alignment records were converted to the Binary Sequence Alignment/Map (BAM) Format with SAMtools [33]. BAM files were sorted twice with Novosort (http://www.novocraft.com/products/novosort/). First, reads were sorted by alignment positions and duplicated read pairs were flagged. Then, reads were sorted by name to group read pairs with identical barcodes together. After sorting, BAM files were converted into BEDPE format using BEDTools [34]. Duplicated and supplementary alignments were ignored. Only read pairs with a mapping quality ≥ 20 were retained. Read pairs with an estimated insert size above 800 bp were discarded. Read pairs were written into a table with four columns (chromosome, start, end, barcode), which was sorted by barcode and alignment position using GNU sort. Read pairs having the same barcode and mapping within 500 kb of each other were assigned to the same molecule. A table recording the barcodes as well as the start and end points of each molecule was exported as a text file. Molecules shorter than 1 kb were discarded.

### 10X super-scaffolding

A graph structure is constructed in which nodes are scaffolds and edges are drawn between if a sufficient number of 10X links meet certain criteria. Only molecules mapping within 100 kb of the scaffold ends are considered. Edges between scaffolds are accepted only if they are supported by molecules from more than one Chromium library. In the initial graph, only molecules connecting scaffolds anchored by POPSEQ to the same chromosome within 5 cM of each other are allowed. A minimum spanning tree is computed with functionalities of the R package igraph [75]. Subsequently, heuristics are applied to resolve branches to obtain subgraphs that are paths. Heuristics include (i) the removal of tips (nodes of rank one), (ii) nodes corresponding to small (< 10 kb) scaffolds, and (iii) cutting the tree at branch points by removing edges. These subgraphs are the initial super-scaffolds, and the order of scaffolds in them is determined by the (well-defined) path traversing each subgraph. The orientation of a scaffold S within a super-scaffold X is determined by calculating the mean position of molecules linking S to other scaffolds in X up to five bins upstream (to the left) or downstream (to the right) of S. If the average position of downstream links is larger than that of the upstream links, the orientation of S is “forward,” and “reverse” otherwise. Super-scaffolds are assigned to POPSEQ genetic positions by lifting positional information from their constituent scaffolds (see below) and computing a consensus. Super-scaffold construction is repeated with different thresholds for the minimum number of read pairs to assign molecules to scaffolds (2 to 10) and the minimum number of molecules required to accept edges between scaffold pairs (2 to 10). Finally, the assembly with the highest N50 is selected for further steps.

### Hi-C map construction

Scaffolds after the GapCloser step were digested in silico with EMBOSS restrict [35]. Reads were aligned to the scaffolds and assigned to restriction fragments as described by Beier et al. [46]. In constrast to Beier et al., we used Minimap2 [32] instead of BWA-MEM [76] for read alignment. Scaffolds were assigned to chromosomes using the POPSEQ genetic maps of wheat and barley [12, 13] as described by Avni et al. [8]. POPSEQ marker sequences (the scaffolds of synthetic wheat W7984 assembled by Chapman et al. [13] or the WGS contigs of the International Barley Sequencing Consortium [52]) were aligned to the scaffolds using Minimap2 [32]. POPSEQ positional information and Hi-C links were lifted from scaffolds to super-scaffolds. Super-scaffolds were ordered and oriented as described by Beier et al. [46]. Intra-chromosomal Hi-C matrices were normalized with HiCNorm [77] and visually inspected in a locally installed R Shiny app. The code for the Shiny app is provided in the Bitbucket repository.

### Discovery and correction of mis-assemblies

We follow the approach of Putnam et al. [54] and Ghurye et al. [45] by detecting sequence mis-joins made during the scaffold stage by inspecting the physical coverage with either 10X molecules or Hi-C links. To find breakpoints based on 10X (Hi-C) data, scaffolds were divided into 200 bp (1 kb) bins and the number of molecules (Hi-C links) spanning each bin was calculated. Link counts were normalized by distance from the scaffold ends. Drops in 10X coverage below one eighth of the genome-wide average were considered as breakpoints. The minimum distance between two breakpoints was 50 kb. After breaking chimeras, POPSEQ marker positions, 10X molecule boundaries, and Hi-C links were lifted to the corrected assemblies. This procedure was repeated until no breakpoints were detected. Drops in Hi-C coverage below 1/16 of the genome-wide average were considered as potential breakpoints, and diagnostic plots summarizing POPSEQ marker information as well as Hi-C coverage along scaffolds were visually inspected. If necessary, breakpoint coordinates were adjusted manually.

### Dovetail assembly

Chicago libraries were prepared from leaves of barley cv. Morex by Dovetail Inc. as described by Putnam et al. [54]. The HiRise algorithm was used to scaffold the non-redundant BAC-based sequence contigs assembled by Mascher et al. [3] (accessible from 10.5447/IPK/2016/30).

### Optical map alignment

The genome-wide optical map of barley cv. Morex [3] was retrieved from 10.5447/IPK/2016/31 and aligned to the TRITEX super-scaffolds using Bionano RefAligner (https://bionanogenomics.com). Custom scripts were used for importing alignments into R and visualization (https://bitbucket.org/tritexassembly/tritexassembly.bitbucket.io/src/master/miscellaneous/bionano.R).

### Assembly-to-assembly alignment

Recent versions of Minimap2 ([32], https://github.com/lh3/minimap2) were used for assembly-to-assembly alignment. Alignment records were written to PAF format and imported into R for visualization and calculation of summary statistics.

### Transcript alignment

Transcript datasets were aligned with GMAP [50] version 2018-07-04 to genomic references. Alignment records were written to GFF files, from which coverage and alignment identity for mRNA alignments were extracted.

### Barley gene annotation

Our annotation pipeline combines three types of evidence for structural gene annotation in plants: protein homology, expression data, and ab initio prediction. For homology-based annotation, we combined available Triticeae protein sequences obtained from UniProt (May 10, 2016). These protein sequences were mapped to the nucleotide sequence of the Morex V2 pseudomolecules using the splice-aware alignment software GenomeThreader [78] (version 1.7.1; arguments -startcodon -finalstopcodon -species rice -gcmincoverage 70 -prseedlength 7 -prhdist 4). Full-length cDNA [42] and IsoSeq [3] nucleotide sequences were aligned to the Morex V2 pseudomolecules using GMAP [50] (version 2018-07-04, standard parameters). Illumina RNA-seq datasets were first mapped using Hisat2 [79] (version 2.0.4, parameter --dta) and subsequently assembled into transcript sequences by Stringtie [80] (version 1.2.3, parameters -m 150 -t -f 0.3). Full-length cDNA, IsoSeq sequences, and RNASeq datasets are described in [3]. All transcripts from flcDNA, IsoSeq, and RNASeq were combined using Cuffcompare [81] (version 2.2.1) and subsequently merged with Stringtie (version 1.2.3, parameters --merge -m 150) to remove fragments and redundant structures. Next, we used Transdecoder (version 3.0.0, https://github.com/TransDecoder) to find potential open reading frames and to predict protein sequences. We used BLASTP [82] (ncbi-blast-2.3.0+, parameters -max_target_seqs 1 -evalue 1e-05) to compare potential protein sequences with a trusted set of reference proteins (Uniprot Magnoliophyta, reviewed/Swissprot, downloaded on 3 August 2016) and used hmmscan [83] (version 3.1b2) to identify conserved protein family domains for all potential proteins. BLAST and hmmscan results were fed back into Transdecoder-predict to select the best translations per transcript sequence.

An independent ab initio annotation using Augustus [84] (version 3.3.2) was carried out to further improve structural gene annotation. To minimize overprediction, hint files using the abovementioned IsoSeq, flcDNA, RNASeq, protein evidences, and TE predictions were generated. The wheat model was used for prediction.

All structural gene annotations were joined by feeding them into EVidenceModeler [85], and weights were adjusted according to the input source. To refine gene models, we also incorporated the barley reference transcript database (BaRT) as an additional source. All BaRT transcripts were aligned to the new Morex V2 assembly using GMAP (version 2018-07-04), and output was converted into GFF format and subsequently fed into EVidenceModeler.

Finally, redundant protein sequences were removed to form a single non-redundant candidate dataset. To categorize candidates into complete and valid genes, non-coding transcripts, pseudogenes, and transposable elements, we applied a confidence classification protocol. Candidate protein sequences were compared against the following three manually curated databases using BLAST: first, PTREP, a database of hypothetical proteins that contains deduced amino acid sequences in which, in many cases, frameshifts have been removed, which is useful for the identification of divergent TEs having no significant similarity at the DNA level; second, UniPoa, a database comprised of annotated Poaceae proteins; and third, UniMag, a database of validated magnoliophyta proteins. UniPoa and UniMag protein sequences were downloaded from Uniprot and further filtered for complete sequences with start and stop codons. Best hits were selected for each predicted protein to each of the three databases. Only hits with an *E* value below 10^−10^ were considered.

Furthermore, only hits with subject coverage (for protein references) or query coverage (transposon database) above 75% were considered significant and protein sequences were further classified using the following confidence: a high confidence (HC) protein sequence is complete and has a subject and query coverage above the threshold in the UniMag database (HC1) or no blast hit in UniMag but in UniPoa and not TREP (HC2); a low confidence (LC) protein sequence is not complete and has a hit in the UniMag or UniPoa database but not in TREP (LC1), or no hit in UniMag and UniPoa and TREP but the protein sequence is complete.

The tag REP was assigned for protein sequences not in UniMag and complete but with hits in TREP.

Functional annotation of predicted protein sequences was done using the AHRD pipeline (https://github.com/groupschoof/AHRD). Completeness of the predicted gene space was measured with BUSCO [55] (version 3.02, orthodb9).

### Analysis of 5′ and 3′ flanking regions of gene models

5′ and 3′ flanking nucleotide sequences of increasing lengths in the range from 1 to 10 kb upstream and downstream of predicted gene models were extracted, and sequences containing N were discarded. The remaining N-free sequences were counted and plotted as a percentage of the total number of predicted gene models.

### Repeat annotation

Transposons were detected and classified by an homology search against the REdat_9.7_Triticeae subset of the PGSB transposon library [86] using vmatch (http://www.vmatch.de) using the following parameters: identity ≥ 70%, minimal hit length 75 bp, and seed length 12 bp (exact command line: -d -p -l 75 -identity 70 -seedlength 12 -exdrop 5). The vmatch output was filtered for redundant hits via a priority-based approach, which assigns higher scoring matches first and either shortens (< 90% coverage and ≥ 50 bp rest length) or removes lower scoring overlaps, leading to an overlap-free annotation.

Full-length LTR retrotransposons were identified with LTRharvest [87] using the following parameters: “overlaps best -seed 30 -minlenltr 100 -maxlenltr 2000 -mindistltr 3000 -maxdistltr 25000 -similar 85 -mintsd 4 -maxtsd 20 -motif tgca -motifmis 1 -vic 60 -xdrop 5 -mat 2 -mis -2 -ins -3 -del -3.” All candidates from the LTRharvest output were subsequently annotated for PfamA domains using hmmer3 [88] and stringently filtered for false positives by several criteria, the main ones being a lack of at least one typical retrotransposon domain (e.g., reverse transcriptase (RT), RNase H (RH), integrase (INT), protease (PR)) and a tandem repeat content > 25%. The inner domain order served as a criterion for the classification into the Gypsy (RT-RH-INT) or Copia (INT-RT-RH) superfamily abbreviated as RLG and RLC. Elements missing either INT or RT were classified as RLX. The insertion age of each full-length LTR retrotransposon was estimated based on the accumulated divergence between its 5′ and 3′ long terminal repeats and a mutation rate of 1.3 × 10^−8^ [89].

Tandem repeats were identified with the TandemRepeatFinder [90] using default parameters and subjected to an overlap removal as decribed above, prioritizing longer and higher scoring elements. K-mer frequencies were calculated with Tallymer [91].

### Representation of selected TE families

We identified full-length copies belonging to single TE families in the assemblies of Chinese Spring and Morex. Our pipeline uses BLASTN [82] to search for long terminal repeats (LTRs) that occur at a user-defined distance range in the same orientation. For RLC_BARE1 and RLC_Angela elements, the two LTRs had to be found within a range of 7800–9300 bp (a consensus RLC_BARE1 sequence has a length of approximately 8700 bp), while a range from 6000 to 10,000 bp was allowed for RLG_Sabrina elements. Multiple different LTR consensus sequences were used for the searches in order to cover the intra-family diversity. Five LTR consensus sequences each were used for RLC_BARE1 and RLG_Sabrina elements, while 18 LTR consensus sequences were used for RLC_Angela elements (to cover the much wider diversity of this family in the 3 wheat subgenomes). The LTR consensus sequences from the same families are 73–92% identical to each other, reflecting the considerable intra-family diversity.

For the current analysis, full-length copies of RLC_BARE1 and their wheat homologs RLC_Angela elements were extracted because these are the most abundant families in barley and wheat genomes, respectively. RLG_Sabrina was chosen because preliminary analyses showed that this TE family has not been active in wheat for a long time and thus is represented mostly by old copies.

To validate the extracted TE populations, the size range of all isolated copies as well as the number of copies that are flanked by a target site duplication (TSD) was determined. A TSD is accepted if it contains at least three matches between 5′ and 3′ TSD (e.g., ATGCG and ACGAG). This low stringency was applied because our previous study showed that TSD generation is error-prone, and thus, multiple mismatches can be expected [92]. In a survey, 80–90% of all isolated full-length elements were flanked by a TSD.

Our pipeline also extracts the so-called solo-LTRs. These are products of intra-element recombination that results in the loss of the internal domain and the generation of a chimeric solo-LTR sequence. Solo-LTRs were used as a metric of how well short repetitive sequences are assembled.

### Data availability

Source code of the TRITEX pipeline is maintained in a Bitbucket repository [93]. A source version as of September 16, 2019 (commit 7041ff2), is accessible under doi: 10.5447/IPK/2019/19 [94]. Paired-end and mate-pair data for Zavitan [8] and Chinese Spring [4] were retrieved from EMBL ENA (accession numbers: PRJEB31422 and SRP114784). Paired-end, mate-pair, 10X, and Chicago data generated for Morex were deposited under ENA project ID PRJEB31444 [95]. Hi-C data for barley cv. Morex [3] generated by Mascher et al. [3] are available under ENA accession PRJEB14169. Sequence assemblies generated in the present study are accessible under the following Digital Object Identifiers (DOIs) in the Plant Genomics and Phenomics Research Data Repository [96]: doi: 10.5447/IPK/2019/7 (wheat assemblies), doi: 10.5447/IPK/2019/8 (barley Morex V2 assembly), and doi: 10.5447/IPK/2019/6 (barley Dovetail assembly). DOIs were registered with e!DAL [97]. TRITEX assemblies are also accessible from EMBL ENA under the following accessions: CACRSD010000000 (Zavitan assembly [98]), CACRSF010000000 (Chinese Spring assembly [99]), LR722616-LR722623 (Morex V2 pseudomolecules [100]), and CABWKO010000000 (barley Dovetail assembly [101]).

### Software versions and documentation of source code

Z shell scripts were used to call assembly and alignment software. GNU Parallel [102] was used for parallel computing. Scaffolding with 10X and Hi-C data was implemented in GNU AWK (https://www.gnu.org/s/gawk/manual/gawk.html) and R [43] scripts. R code relies heavily on the data.table package (https://cran.r-project.org/package=data.table) for in-memory data management and analysis. A step-by-step usage guide was prepared using the AsciiDoc markup language (https://asciidoctor.org/docs/what-is-asciidoc/) and is available from https://nomagicassembly.bitbucket.io. A list of software versions known to work with TRITEX is included in the usage guide. Assemblies were run on compute servers at IPK Gatersleben. The most powerful of these has 72 physical cores (Intel Xeon E7-8890 v3) and 2 TB of main memory. We had access to 100 TB of hard disk space and 22 TB of SSD storage.

## Supplementary information

**Additional file 1.** Supplementary Tables and Figures.

**Additional file 2.** Review History.
