# Supplementary material for: TRITEX: chromosome-scale sequence assembly of Triticeae genomes with open-source tools
Source: Genome Biol. 2019 Dec 18;20:284. doi: 10.1186/s13059-019-1899-5 (PMC6918601; doi:10.1186/s13059-019-1899-5)
Supplement: Supplementary file 2 — Additional file 2. Review History. [file 13059_2019_1899_MOESM2_ESM.docx]

Review History

**First round of review**

**Reviewer 1**

**Comments to author:**

This manuscript by Monat (Mascher) and colleagues describes a new enabling tool, TRITEX, for assembly of complex genomes from short read (Illuminna) sequencing data. The method was tested out on barley (a diploid) and bread wheat (a hexaploid) and shown to perform to a comparable high standard as the commercial (not open source) method, DENOVO MAGIC, developed by the company NRGene.

The announcement about three years ago by NRGene that they could assemble the complex wheat genome into megabase-sized scaffolds from short read Illumina data generated quite a stir in the community, with many observers and scholars initially claiming that this was "hocus pocus" and that the method relied on already existing data (e.g. alignment to barley) to achieve the high contiguity and large scaffolds. Ever since, there has been a fury of activity in the plant genome community to develop an open source method that could achieve similar outputs as NRGene's DENOVO MAGIC. The present study unequivocally delivers such a method. Central to this method, dubbed TRITEX, is the generation of overlapping 250 bp PE reads which allow an initial assembly with a very long k-mer size.

I expect that the TRITEX pipeline will become widely adopted by the community in years to come, and that at least parts of it will supersede the advent of long read technologies. I expect the manuscript will be of great interest to a wide readership working on plant and animal genomics. I have no hesitation in endorsing the manuscript for publication in Genome Biology. I only picked up on a few very, very minor typos when reading the manuscript:

line 71. Please correct grammar
line 343. Please correct grammar
Line 385. Alternatives should be singular.
Line 467: Should be 700 - 800 bp (not kb).
Lines 561-2: Please correct grammar.
Line 692: should be: "... using the following parameters"

**Reviewer 2**

**Comments to author:**

This manuscript describes an open-source pipeline, TRITEX, for de novo assembly of Triticeae genomes at the chromosome scale. Using this pipeline, the authors assembled an improved version of the barley cv. Morex genome using the newly generated high-throughput Illumina and 10x Chromium sequencing data. Developing novel open-source tools for efficient assembly of large and complex genomes of plant species such as those of Triticeae will for sure facilitate genome-based discovery and genome-assisted breeding in these crops. The improved assembly of the barley genome provides a valuable resource for the plant community.

Major:
1.     The biggest concern I have is that the TRITEX pipeline the authors developed only provides incremental conceptual and technical advances. The authors just put together the commonly used open-source tools for sequence processing and assembly into one single pipeline. The pipeline to me has nothing new or innovative for Triticeae genome assemblies. As genome assemblies are mainly conducted by experienced bioinformaticians, running the individual programs included in the TRITEX pipeline, as well as other commonly used programs for read processing (e.g., Trimmomatic) or assembly (e.g., MaSuRCA), should not be an issue. Therefore, the interest of the TRITEX pipeline by the plant research community could be limited.
2.     The authors did not explain the uniqueness of Triticeae genomes, and why the TRITEX pipeline can be used for the assembly of the Triticeae genomes. The authors did not compare other assemblers to the ones used in the pipeline (Minia3 for assembly and SOAPDenovo2 for scaffolding), and the only reason Minia3 was included in TRITEX is that Minia3 can use k-mers of arbitrary sizes. However, this does not mean that Minia3 is better than other assemblers in assembling Triticeae genomes.
3.     Line 387-395: The application of TRITEX to genome assemblies of different species described here lacks data support. The authors need to explain why TRITEX can be applied to "diploid or allopolyploid inbreeding Triticeae species", rye, and "large and allopolyploid genomes outside the Triticeae tribe such as maize and oats".
4.     I suggest that the authors shift the focus of this manuscript from the TRITEX pipeline to the improved genome assembly of the barley cv. Morex. Most of the first section of the "Results" then can go to the "Methods" section and the second section can be put in supplementary note. The improved Morex genome assembled from the newly generated Illumina paired-end and mate-pair data as well as 10x Chromium data provides a very important resource and should have much broader interest than the TRITEX pipeline. Of course, additional analyses of the improved genome to derive interesting biological insights would strengthen the manuscript.

Minor:
5.     Line 71: "assembly" to "assembled".
6.     Line 93-94, "compare our assemblies to the those constructed": remove "the".
7.     Line 204: remove "in comparison"
8.     References 4 and 24 are duplicated, same for references 8 and 17, and 41 and 69. Please check the entire reference section carefully (e.g., author names for reference 91 seems weird).

**Authors Response**

**Point-by-point responses to the reviewers’ comments:**

Reviewer #1: This manuscript describes an open-source pipeline, TRITEX, for de novo assembly of Triticeae genomes at the chromosome scale. Using this pipeline, the authors assembled an improved version of the barley cv. Morex genome using the newly generated high-throughput Illumina and 10x Chromium sequencing data. Developing novel open-source tools for efficient assembly of large and complex genomes of plant species such as those of Triticeae will for sure facilitate genome-based discovery and genome-assisted breeding in these crops. The improved assembly of the barley genome provides a valuable resource for the plant community.

Major:
1. The biggest concern I have is that the TRITEX pipeline the authors developed only provides incremental conceptual and technical advances. The authors just put together the commonly used open-source tools for sequence processing and assembly into one single pipeline. The pipeline to me has nothing new or innovative for Triticeae genome assemblies. As genome assemblies are mainly conducted by experienced bioinformaticians, running the individual programs included in the TRITEX pipeline, as well as other commonly used programs for read processing (e.g., Trimmomatic) or assembly (e.g., MaSuRCA), should not be an issue. Therefore, the interest of the TRITEX pipeline by the plant research community could be limited.

*Response: We agree with the reviewer that our paper does not present a novel algorithm, but is a combination of existing tools. We believe that it is more than an incremental conceptual and technical advance to develop and evaluate a working “recipe” for assembling the complex genomes of Triticeae species. We describe which datasets to generate and which programs to use with which parameters to achieve a chromosome-scale assembly. By comparison to published data, we show that TRITEX performs similar to commercial alternatives and better than open-source alternatives.*

*Genome assembly is not only performed in frame of dedicated genome projects, but also used as a tool for more targeted applications (diversity studies, map-based cloning projects – see our Discussion). Thus, the bioinformatics researchers involved in these projects may be familiar with tasks such as read mapping, variant calling or statistical analysis in R, but may be overwhelmed with the plethora of assembly tools available. After the public release of the source code and the tutorial on Bitbucket (several months before submitting the paper), we have received positive feedback on the level of detail.

We have added a note in the Discussion section (ll. 428-432) of the revised manuscript that several tasks for the assembly workflow (such as trimming and error correction) can also be performed with other tools. However, we are convinced that the use of Minia3 for contig assembly is an integral part of our pipeline to achieve good genome representation and ensure the scalability of TRITEX to pan-genome projects with moderate computational resources (see our response to the next point).*

2. The authors did not explain the uniqueness of Triticeae genomes, and why the TRITEX pipeline can be used for the assembly of the Triticeae genomes. The authors did not compare other assemblers to the ones used in the pipeline (Minia3 for assembly and SOAPDenovo2 for scaffolding), and the only reason Minia3 was included in TRITEX is that Minia3 can use k-mers of arbitrary sizes. However, this does not mean that Minia3 is better than other assemblers in assembling Triticeae genomes.

*Response: The use of large k-mers (and hence the use of Minia3) is an integral part of our pipeline. We have stated this explicitly in the revised Discussion (ll. 429-430). We have added a new supplementary table (Table S1) to show the effect of using large k-mers. Even a k-mer size of 200 results in a larger fraction of the genome that is not assembled. At least, in the Triticeae larger k-mer sizes may be necessary to resolve a sufficiently large fraction of the repetitive space to enable efficient scaffolding with mate-pair reads.
TRITEX is a modular pipeline and we agree that other tools maybe used to perform tasks such as trimming, error correction and scaffolding. We have added this statement to the Discussion section (ll. 430-432). During the development of the pipeline, we had tried different scaffolding tools (SOAPDenovo2, BESST, OPERA-LG). We have added a new supplementary table (Table S2) and describe it in ll. 139-142 of the Results section.*

3. Line 387-395: The application of TRITEX to genome assemblies of different species described here lacks data support. The authors need to explain why TRITEX can be applied to "diploid or allopolyploid inbreeding Triticeae species", rye, and "large and allopolyploid genomes outside the Triticeae tribe such as maize and oats".

*Response: In collaborative projects, we have run TRITEX on other species, or advised researchers how to use TRITEX. After consulting with the editor, we decided not to add new datasets and a full description of these assemblies, but only to guide potential users which assembly results to expect in other species. The raw data, assembly and analyses based on it will be reported in separate publications. The first of these are expected to be released as BioRxiv pre-prints in the coming months.
In the revised manuscript, we have expanded the Discussion section (ll. 394-415) to present three lines of evidence why TRITEX will work as well in other species:
1. We used the full TRITEX pipeline including 10X and Hi-C data for assembling chromosome-scale reference sequences of the diploid wild relatives Ae. sharonensis and Ae. longissima. These sequence assemblies will form the basis of a comparative genomics paper to study the relationship of these genomes with bread wheat subgenomes. Summary statistics of these assemblies are presented in the revised manuscript.
2. We have evaluated the performance of TRITEX in hexaploid oat (Avena sativa). We used raw data paired-end and mate-pair and achieved a scaffold N50 values that is similar to that of our bread wheat assembly.
3. We have not yet used TRITEX in maize. However, several reports on assemblies with NRGene’s DeNovoMagic software have been published recently (Hirsch et al., 2016, Plant Cell; Unterseer et al. 2017, BioRxiv; Springer et al, Nature Genetics 2018). As the contiguity of TRITEX and DeNovoMagic assemblies is comparable in bread wheat, it is reasonable to expect similar results in maize.*

4. I suggest that the authors shift the focus of this manuscript from the TRITEX pipeline to the improved genome assembly of the barley cv. Morex. Most of the first section of the "Results" then can go to the "Methods" section and the second section can be put in supplementary note. The improved Morex genome assembled from the newly generated Illumina paired-end and mate-pair data as well as 10x Chromium data provides a very important resource and should have much broader interest than the TRITEX pipeline. Of course, additional analyses of the improved genome to derive interesting biological insights would strengthen the manuscript.

*Response: We agree that the value of the present paper lies not only in describing the TRITEX assembly pipeline, but also in presenting an updated reference sequence assembly of the barley genome as an important community resource. We have used TRITEX for other species and have received positive feedback from the community for providing an open-source alternative to NRGene’s DeNovoMagic assembly service (see also the comments of Referee 2). Thus, we are convinced that TRITEX will not only be applied in barley and wheat pan-genomics projects, but also in other crop species and wild relatives. Following the editor’s advice, we have kept the focus of the manuscript on presenting the TRITEX assembly pipeline.*

Minor:
5. Line 71: "assembly" to "assembled".
6. Line 93-94, "compare our assemblies to the those constructed": remove "the".
7. Line 204: remove "in comparison"
8. References 4 and 24 are duplicated, same for references 8 and 17, and 41 and 69. Please check the entire reference section carefully (e.g., author names for reference 91 seems weird).

*Response: We have corrected these editorial issues.*


Reviewer #2: This manuscript by Monat (Mascher) and colleagues describes a new enabling tool, TRITEX, for assembly of complex genomes from short read (Illuminna) sequencing data. The method was tested out on barley (a diploid) and bread wheat (a hexaploid) and shown to perform to a comparable high standard as the commercial (not open source) method, DENOVO MAGIC, developed by the company NRGene.

The announcement about three years ago by NRGene that they could assemble the complex wheat genome into megabase-sized scaffolds from short read Illumina data generated quite a stir in the community, with many observers and scholars initially claiming that this was "hocus pocus" and that the method relied on already existing data (e.g. alignment to barley) to achieve the high contiguity and large scaffolds. Ever since, there has been a fury of activity in the plant genome community to develop an open source method that could achieve similar outputs as NRGene's DENOVO MAGIC. The present study unequivocally delivers such a method. Central to this method, dubbed TRITEX, is the generation of overlapping 250 bp PE reads which allow an initial assembly with a very long k-mer size.

I expect that the TRITEX pipeline will become widely adopted by the community in years to come, and that at least parts of it will supersede the advent of long read technologies. I expect the manuscript will be of great interest to a wide readership working on plant and animal genomics. I have no hesitation in endorsing the manuscript for publication in Genome Biology. I only picked up on a few very, very minor typos when reading the manuscript:

line 71. Please correct grammar
line 343. Please correct grammar
Line 385. Alternatives should be singular.
Line 467: Should be 700 - 800 bp (not kb).
Lines 561-2: Please correct grammar.
Line 692: should be: "... using the following parameters"

*Response: We have corrected these errors.*

**Second round of review**

**Reviewer 1**

The authors have addressed my concerns
